# Merrell, Thorp & Loyd, Cincinnati

**Published:** 1877-05-20

**Authors:** 


MERRELL, THORP & LOYD, CINCINNATI.—This house comes highly recommended to us as pharmacists and manufacturing chemists. See thier card elsewhere.

